# A combination pharmacotherapy of tapentadol and pregabalin to tackle centrally driven osteoarthritis pain

**DOI:** 10.1002/ejp.1386

**Published:** 2019-03-22

**Authors:** Stevie Margaret Lockwood, Anthony H. Dickenson

**Affiliations:** ^1^ Department of Neuroscience, Physiology and Pharmacology University College London London UK

## Abstract

**Background:**

Many Osteoarthritis (OA) patients report with clinical features to their pain that cannot be explained by purely peripheral mechanisms. Yet, the analgesic agents available that tackle centrally driven chronic pain often provide only partial pain relief, or have dose‐limiting side effects. We explored a combination therapy of the centrally acting analgesic agents tapentadol and pregabalin, to investigate if they could be used in combination to provide superior analgesia.

**Methods:**

Using electrophysiological single‐unit recordings taken from spinal wide dynamic range neurons, Diffuse Noxious Inhibitory Controls (DNIC) were assessed as a marker of potential changes in descending controls in a monoiodoacetate (MIA) model of OA. We investigated if a subcutaneous injection of tapentadol or pregabalin, both alone and in combination, inhibited neuronal responses and restored the expression of DNIC, quantified as a reduction in neuronal firing in the presence of a conditioning noxious stimulus.

**Results:**

Tapentadol restored DNIC‐induced neuronal inhibition in MIA animals, while pregabalin inhibited pre‐conditioned mechanically evoked neuronal responses but did not restore DNIC. Given in combination, tapentadol and pregabalin restored DNIC expression and also inhibited spinal neuronal responses.

**Conclusions:**

We propose that there is both central sensitization and an imbalance in inhibitory and facilitatory descending controls in MIA animals. The combination therapy of tapentadol and pregabalin restored descending noradrenergic inhibitory tone and also inhibited nociceptive transmission at the level of the spinal cord.

**Significance:**

This study shows that pregabalin and tapentadol target different mechanisms of centrally driven chronic pain associated with osteoarthritis, and that when administered together can restore descending inhibitory tone whilst also tackling spinal neuronal hyperexcitability and may therefore provide superior analgesia.

## 1 INTRODUCTION

Most analgesics used to treat Osteoarthritis (OA) aim to tackle pain at the level of the joint, yet the continuous barrage of afferent nociceptive signals and the high level of plasticity in the central nervous system (CNS) means hypersensitivity of second‐order neurons in the dorsal horn often develops (Schaible, 2012; Wieland, Michaelis, Kirschbaum, & Rudolphi, 2005). Spinal cord neurons receiving input from the joint are also under the influence of descending controls arising in the brainstem, and impairment of descending controls also contributes to an exaggerated pain response in OA patients (Gwilym et al., 2009). Many OA patients develop referred pain at sites distant to initial joint damage and suffer from chronic pain following total knee replacement surgery, which suggests discordance between nociceptor activation and the resulting pain (Malfait & Schnitzer, 2014). These features indicate that the pain associated with OA cannot always be considered purely peripheral; it is important to consider spinal hypersensitivity and dysfunctional descending controls when tackling these clinical manifestations (Wylde, Hewlett, Learmonth, & Dieppe, 2011). However, the limited treatment options available that target centrally driven chronic pain often provide only partial pain relief or come with substantial side effects (Finnerup & Jensen, 2007).

Tapentadol is a centrally acting analgesic that combines two modes of action; its μ‐opioid receptor (MOR) agonism activates descending opioidergic controls, whilst its Noradrenaline Reuptake Inhibitor (NRI) function increases the synaptic availability of noradrenaline and produces analgesia through the activation of α2‐adrenoceptors (Schroder et al., 2011; Tzschentke, Folgering, Flik, & De Vry, 2012; Tzschentke et al., 2009; Wade & Spruill, 2009). Tapentadol has proven an effective analgesic in OA patients, with better tolerability than traditional opioids (Pergolizzi, Taylor, LeQuang, Raffa, & Bisney, 2018). Functional descending controls and the release of neurotransmitters into the spinal cord, particularly noradrenaline, is crucial for the expression of Diffuse Noxious Inhibitory Controls (DNIC), a unique form of endogenous inhibitory control where the activity of convergent spinal neurons is strongly inhibited by a conditioning noxious stimulus (Bannister, Lockwood, Goncalves, Patel, & Dickenson, 2017; Bannister, Patel, Goncalves, Townson, & Dickenson, 2015; Le Bars, Chitour, Kraus, Dickenson, & Besson, 1981). The human counterpart of DNIC, Conditioned Pain Modulation (CPM), also relies on descending controls, as it is lost in tetraplegics (Roby‐Brami, Bussel, Willer, & Le Bars, 1987). A reduced CPM has been reported in OA patients, strongly indicating dysregulated top‐down modulation develops as the pain state progresses (Arendt‐Nielsen et al., 2010). Tapentadol holds potential as a powerful analgesic molecule as it restores descending inhibitory tone through synergistic interaction between its two distinguished mechanisms (Schroder et al., 2011).

Pregabalin is an analgesic agent preferentially effective in chronic pain states with hypersensitivity of neurons in the CNS (Stahl et al., 2013). Pregabalin binds to the α_2_δ_1_ subunit of voltage‐gated calcium channels (VGCCs) and inhibits calcium currents and the release of neurotransmitters, allowing it to modulate dysregulated neuronal signals (Davies et al., 2007). Pregabalin is particularly effective in neuropathic conditions, and as a subset of OA patients report neuropathic features to their pain, pregabalin holds the potential to solve an unmet analgesic need (Patel & Dickenson, 2016; Thakur, Dickenson, & Baron, 2014).

We used a monoiodoacetate (MIA) model of OA, which mimics clinical manifestations of the human condition, including joint pathology and pain‐like behaviour. We investigated how tapentadol and pregabalin affected spinal neuronal activity and the functionality of descending controls through measuring DNIC responses in MIA animals.

## 2 METHODS

### 2.1 Animals

In all experiments, male Sprague Dawley rats were used. Food and water were provided ab libitum, with cages kept in a 12 hr light/dark cycle. All experiments were performed in accordance with the UK Animals (Scientific Procedures) Act 1986, in terms of project and personal licences following approval by the UCL ethics committee.

### 2.2 The MIA model

Male Sprague Dawley rats (120–140 g) were anaesthetized with isoflurane, and arthritis was induced in the left knee by an intrarticular injection of 2 mg MIA (Sigma, UK) in 25 μl of 0.9% saline. Sham animals received an intrarticular injection of 25 μl 0.9% saline only. The experimenter was blinded as to which substance animals received.

### 2.3 Electrophysiology

Electrophysiological experiments were carried out 14–20 days post‐MIA injection as previously described (Urch & Dickenson, 2003). Briefly, animals were anesthetized for the duration of the experiment with a constant flow of isoflurane (1.5%) delivered via a tracheal cannula in a gaseous mix of O_2_ (33%) and N_2_O (66%). A laminectomy was performed to expose the L4‐L5 segments of the spinal cord. Extracellular single‐unit recordings were made from deep dorsal horn wide dynamic range (WDR) neurons (Lamina V‐VI) using parylene‐coated tungsten electrodes (A‐M systems). All WDR neurons used in this study responded to both innocuous and noxious stimulations of the hind paw in a graded manner coding intensity. Data were captured and analysed by a CED 1401 interface coupled to a computer running Spike2 software (Cambridge Electronic Design; rate functions).

### 2.4 DNIC study design

Firstly, the pre‐conditioned mechanically evoked neuronal firing rates were quantified in response to 8, 26 and 60 g von Frey filament stimulation applied to the hind paw. This was repeated three times to obtain a stable pre‐conditioned response (where all neurons met the inclusion criteria of <10% variation in action potential firing). For the DNIC response, the same von Frey filaments were applied to the receptive field with a concurrent noxious ear pinch (15.75 × 2.3 mm Bulldog Serrefine, Interfocis, Linton). This counted as one trial and pre‐conditioned and DNIC responses were calculated as the mean from two trials. A DNIC response was quantified as an inhibition on mechanically evoked neuronal firing in the presence of the conditioning noxious ear pinch. A 1‐min nonstimulation recovery period was allowed between each test, while a 10‐min nonstimulation recovery period was allowed between each trial to ensure neuronal responses had returned to baseline.

### 2.5 Drug administration

**Figure 1:**
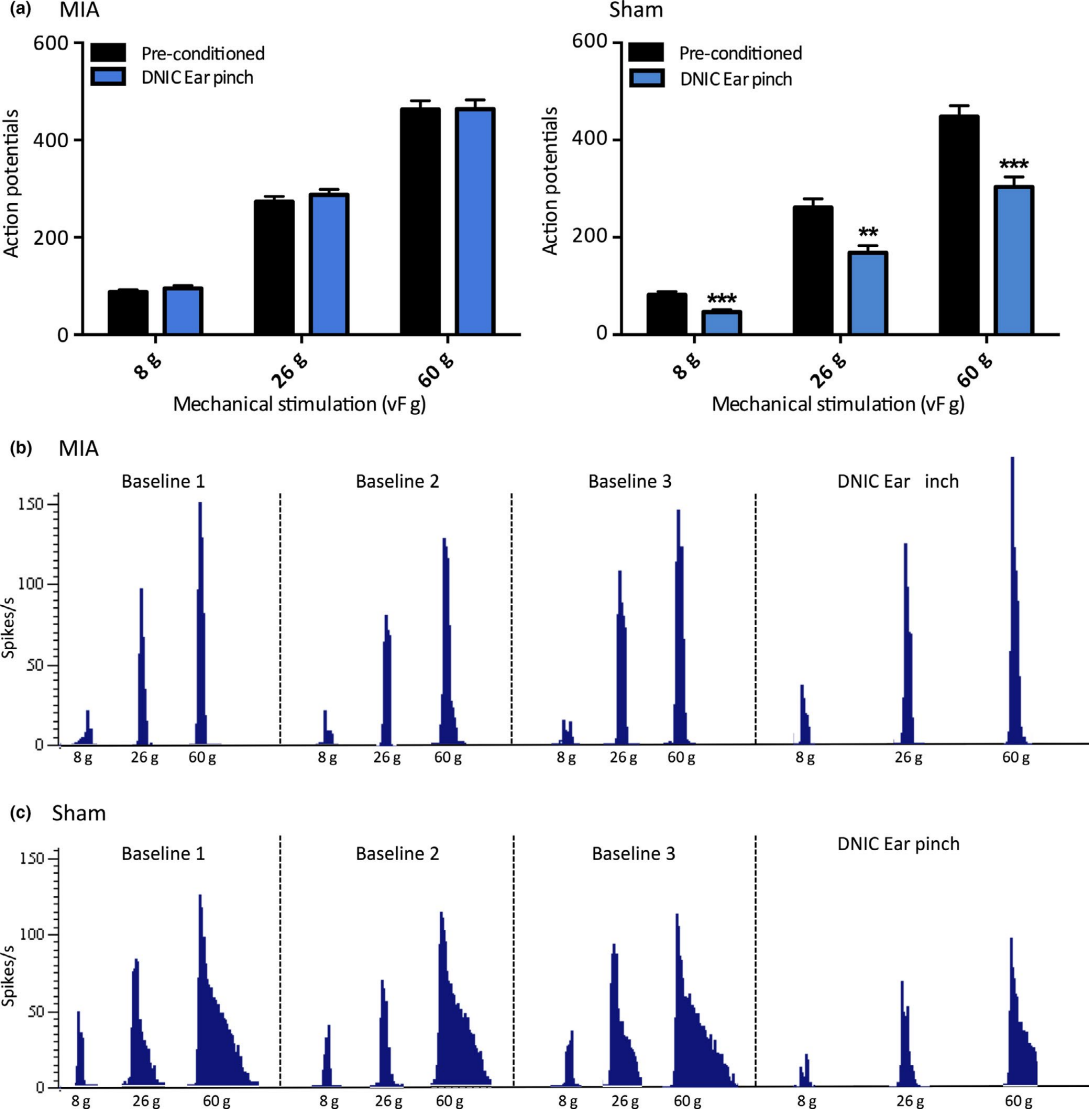
The expression of DNIC in MIA animals (*n* = 42) and sham controls (*n* = 27). The presence of DNIC was confirmed by a reduction in mechanically evoked WDR neuronal firing in the presence of a conditioning noxious ear pinch. (a) A conditioning noxious ear pinch had no significant effect on mechanically evoked neuronal firing in MIA animals. (b) In sham controls, mechanically evoked WDR neuronal responses were significantly inhibited by a noxious conditioning ear pinch. (c) The trace represents a single‐unit recording from a WDR neuron in an MIA animal, showing three baseline pre‐conditioned responses and one DNIC response in the presence of a conditioning noxious ear pinch. There is no reduction in neuronal firing with a concurrent noxious ear pinch. (d) The trace represents a single‐unit recording from a WDR neuron in a sham control animal, with three baseline pre‐conditioned responses and one DNIC response in the presence of a conditioning noxious ear pinch. When the conditioning noxious ear pinch is applied, there is a substantial reduction in neuronal firing in response to mechanical stimulations. Two‐way ANOVA with Bonferroni correction tested statistically significant differences from pre‐conditioned baseline responses; **p < 0.01, ***p < 0.001

**Figure 2:**
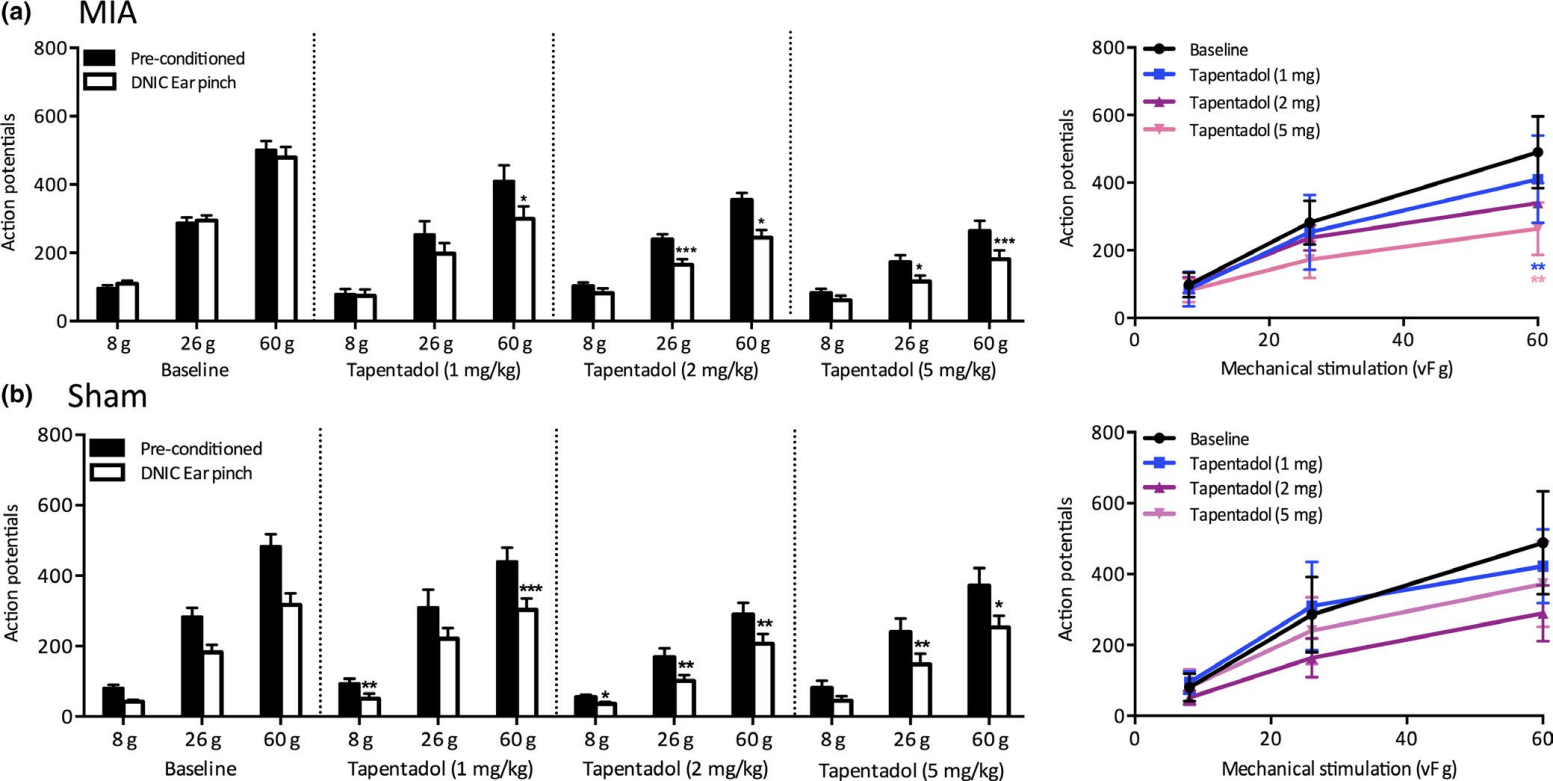
The effect of systemic tapentadol on WDR neuronal responses in MIA and sham animals. (a) Tapentadol restored neuronal inhibition induced by DNIC in MIA animals; for all doses (1 mg/kg *n* = 8, 2 mg/kg *n* = 6, and 5 mg/kg *n* = 7) there was a significant reduction in neuronal firing in response to a 60 g mechanical stimulation in the presence of a conditioning noxious ear pinch, while 2 and 5 mg produced a significant reduction in neuronal firing induced by DNIC in response to a 26 g mechanical stimulation. Tapentadol also dose‐dependently inhibited pre‐conditioned mechanically evoked neuronal responses, but this effect was only significant for the most noxious 60 g stimulation. (b) Tapentadol had little effect on the magnitude of neuronal inhibition induced by DNIC in sham controls as the degree of inhibition remained comparable before and after tapentadol administration for all doses tested (*n* = 6 for all doses). Tapentadol inhibited pre‐conditioned mechanically evoked neuronal responses in sham animals but this effect was not significant for any doses or mechanical forces tested. Two‐way ANOVA with Bonferroni correction tested statistically significant differences from pre‐conditioned baseline responses for both DNIC‐induced and drug‐induced neuronal inhibition; *p < 0.05, **p < 0.01, ***p < 0.001

**Figure 3:**
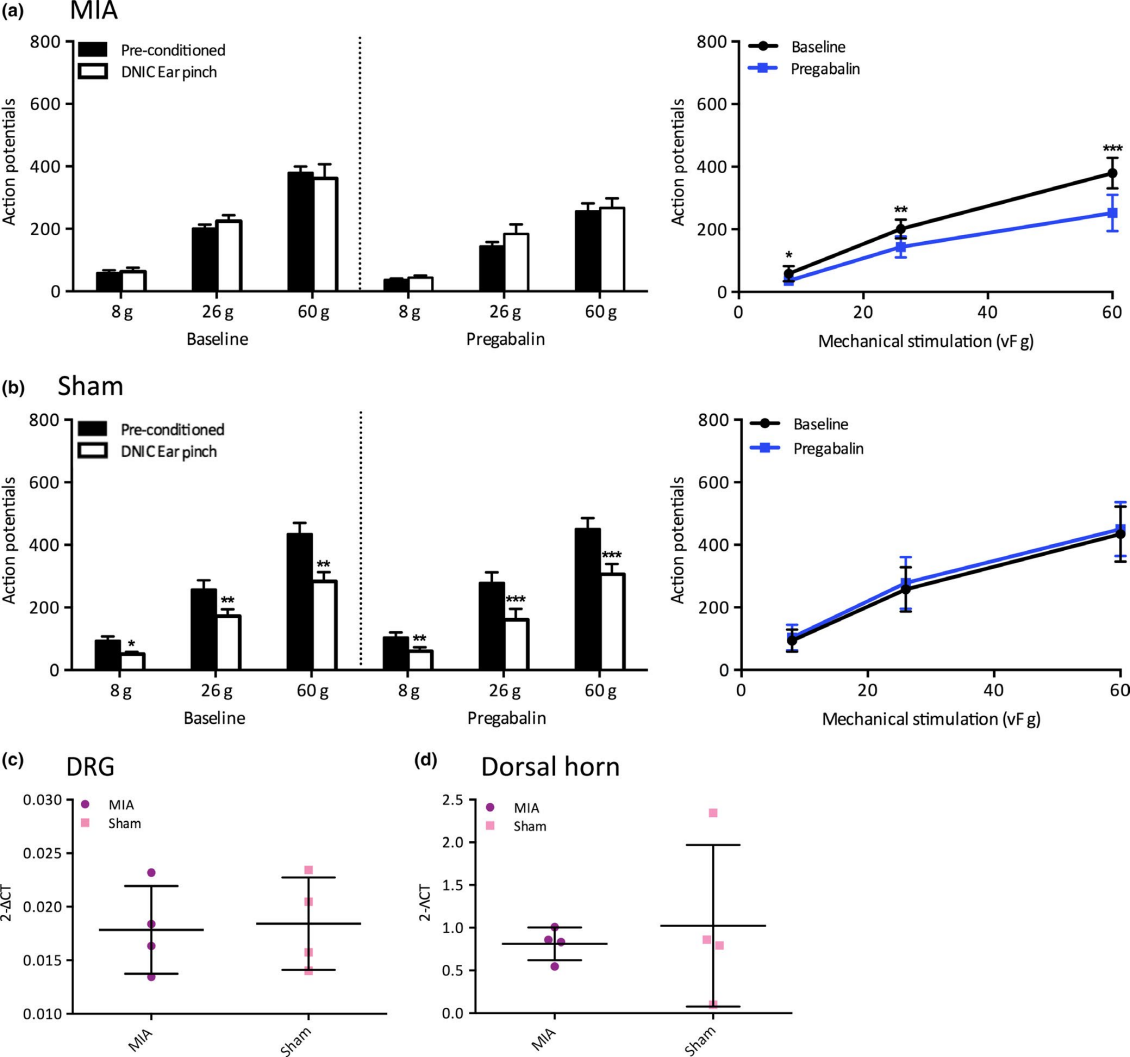
The effect of systemic pregabalin on WDR neuronal responses in MIA and sham animals. (a) Following a subcutaneous injection of pregabalin (10 mg/kg), there was no neuronal inhibition induced by DNIC, yet there was a significant reduction in pre‐conditioned neuronal firing for all mechanical stimulations tested (*n* = 6). (b) Pregabalin had little effect on the degree of neuronal inhibition induced by DNIC in sham controls (*n* = 6) and had no statistically significant effect on mechanically evoked pre‐conditioned neuronal responses. (c–d) There was no significant difference in the mRNA expression levels of the α2δ1 subunit in either ipsilateral lumbar DRGs or dorsal horn. Two‐way ANOVA with Bonferroni correction tested statistically significant differences from pre‐conditioned baseline responses for both DNIC‐induced and drug‐induced neuronal inhibition, independent samples *t* test tested statistically significant differences in mRNA expression levels; *p < 0.05, **p < 0.01, ***p < 0.001

**Figure 4:**
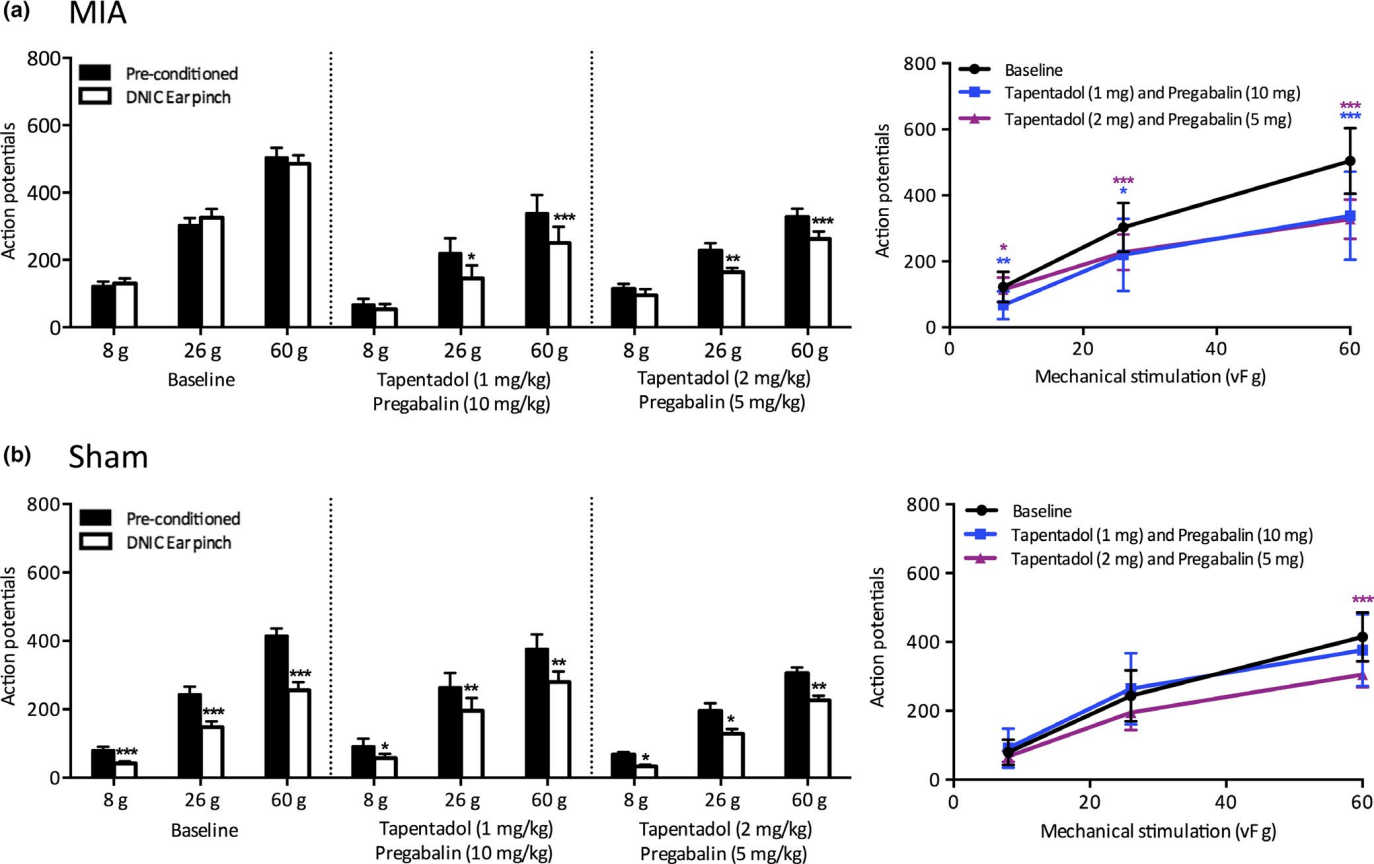
The effect of a combination pharmacotherapy of tapentadol and pregabalin on WDR neuronal responses in MIA and sham animals. (a) In MIA animals, both dose combinations produced a significant reduction in neuronal firing induced by DNIC in response to 26 and 60 g mechanical stimulations and significantly inhibited pre‐conditioned neuronal firing for all mechanical stimulations (*n* = 6 for both doses). (b) In sham controls, both dose combinations had little effect on the magnitude of neuronal inhibition induced by DNIC. The 1 mg tapentadol and 10 mg pregabalin dose combination had no significant effect on pre‐conditioned mechanically evoked neuronal firing (*n* = 6). The 2 mg tapentadol and 5 mg pregabalin dose significantly inhibited pre‐conditioned neuronal firing in response to a 60 g stimulation, which can likely be attributed to the higher tapentadol dose (*n* = 5). Two‐way ANOVA with Bonferroni correction tested statistically significant differences from pre‐conditioned baseline responses for both DNIC‐induced and drug‐induced neuronal inhibition; *p < 0.05, **p < 0.01, ***p < 0.001

Firstly, two DNIC trials were carried out to collect pre‐drug baseline controls. Each individual drug dose was then administered, and the neuronal response was followed for one hour, with tests carried out at 20 and 40 min (one neuron per animal). For each time point, another DNIC trial was conducted, which consisted of pre‐conditioned responses to 8, 26 and 60 g mechanical stimulations repeated three times to obtain stable responses, followed by a DNIC response with a concurrent noxious ear pinch. For post‐drug effects, the maximal changes for pre‐conditioned and DNIC responses are presented in the graphs for Figures 1, 2, 3, 4.

Tapentadol (Grünenthal, Germany) was dissolved in saline and delivered via a subcutaneous injection at three doses; 1, 2 and 5 mg/kg. Pregabalin (Pfizer, UK) was dissolved in normal saline and delivered via a subcutaneous injection to give a systemic dose of 10 mg/kg. A combination treatment of tapentadol and pregabalin was delivered in the same solution via a subcutaneous injection at two dose combinations: 1 mg/kg tapentadol with 10 mg/kg pregabalin, and 2 mg/kg tapentadol with 5 mg/kg pregabalin. One dose was tested per neuron to avoid accumulative dosing.

### 2.6 qPCR

Animals were terminally anaesthetized with an overdose of isoflurane, and the ipsilateral lumbar dorsal horn and L3‐L5 DRGs were dissected, snap frozen in liquid nitrogen and stored at −80°C. RNA was extracted from homogenized tissue using a RNAse microkit (Qiagen). First strand cDNA synthesis was performed on 500 ng RNA using a Superscript III Reverse Transcriptase kit (Invitrogen) according to manufacturers instructions with deoxynucleotide‐triphosphates (Promega) and random primers (Promega). mRNA levels of the α_2_δ_1_ subunit were measured with quantitative PCR using specific primers (Forward: CATTGTTGGGCTCCACAGTAT, Reverse: GACCTTGTCACACTGGCAAA) and LightCycler^®^ 480 SYBR Green I master mix (Roche, UK). The mRNA levels were normalized to GAPDH and expressed as either 2‐ΛCT values.

### 2.7 Statistical analysis

Statistical analyses were performed using SPSS v22 (IBM, Armonk). All data plotted represent the mean ± *SEM*. For electrophysiology, statistical differences in neuronal responses between pre‐conditioned and DNIC responses, or following drug application were determined using a two‐way repeated‐measures ANOVA with Bonferroni post hoc test. For qPCR, statistical differences in mRNA expression levels between MIA and sham controls were determined with an independent sample *t* test. Asterisks denote statistically significantly differences (*p < 0.05, **p < 0.01, ***p < 0.001).

## 3 RESULTS

### 3.1 DNIC expression in MIA animals

The presence of DNIC was confirmed by a reduction in mechanically evoked WDR neuronal firing in the presence of the noxious conditioning ear pinch. Saline‐injected sham animals had a normally functioning DNIC system, with the conditioning noxious pinch producing a significant and consistent reduction in mechanically evoked neuronal activity (two‐way ANOVA; 8 g: *p* < 0.001, 26 g: *p* < 0.01, 60 g: *p* < 0.001, *n* = 27) (Figure 1b). The degree of DNIC‐induced inhibition averaged 45% for 8 g, 38% for 26 g and 37% for 60 g, which is comparable to the magnitude reported in naïve animals of a 30%–40% reduction in neuronal firing (Bannister et al., 2015; Le Bars, Dickeonson, & Besson, 1979). However, when the magnitude of DNIC was assessed in MIA animals (>14 days post‐injection), the noxious conditioning ear pinch no longer produced a concurrent inhibition of WDR neuronal firing for any von Frey forces tested (two‐way ANOVA; *p* > 0.05 for all forces, *n* = 42) (Figure 1a).

### 3.2 The actions of tapentadol in MIA animals

The DNIC system relies upon descending noradrenergic inhibitory controls, but has also been reported to have a partly opioidergic component (Bannister et al., 2015, 2017; Le Bars et al., 1981). Therefore, as tapentadol modulates these two descending systems, we assessed the effects of systemic tapentadol on spinal neuronal activity and DNIC expression in MIA and sham animals.

All doses of tapentadol tested (1, 2 and 5 mg/kg) restored the inhibitory effect on mechanically evoked neuronal firing induced by a noxious conditioning ear pinch (Figure 2a). Following systemic doses of tapentadol, the degree of neuronal inhibition induced by a conditioning noxious ear pinch was 6%, 22% and 27% with 1 mg/kg (*n* = 8), 21%, 31% and 31% with 2 mg/kg (*n* = 6), and 25%, 33% and 31% with 5 mg/kg (*n* = 7) for 8, 26 and 60 g mechanical stimulations, respectively. Interestingly, for the 1 mg/kg dose there was only a significant reduction of neuronal firing induced by DNIC in response to a 60 g mechanical stimulation (two‐way ANOVA; 8 and 26 g: *p* > 0.05, 60g: *p*<0.05), while for 2 and 5 mg/kg doses there was a significant reduction of neuronal firing induced by DNIC in response to 26 and 60 g mechanical stimulations (two‐way ANOVA; 2 mg/kg: 8 g: *p* > 0.05, 26 g: *p* < 0.001, 60 g: *p* < 0.05, 5 mg/kg: 8 g: *p* > 0.05, 26 g: *p* < 0.05, 60 g: *p* < 0.001). In addition, tapentadol dose‐dependently reduced pre‐conditioned mechanically evoked neuronal firing, but this was only significant for the most noxious 60 g force (Figure 2a) (two‐way ANOVA: 1 mg/kg: 60 g: *p* < 0.01, 5 mg/kg: 60 g: *p* < 0.01; See Supporting Information Table S1).

The effects of tapentadol on mechanically evoked neuronal firing rates and DNIC expression were also explored in saline‐injected sham control animals. Following all doses of systemic tapentadol, there was a significant reduction in mechanically evoked neuronal firing with a noxious conditioning ear pinch, but there was no consistent effect on the magnitude of neuronal inhibition induced by DNIC (1 mg/kg: *n* = 6, 2 mg/kg: *n* = 6, 5 mg/kg: *n* = 6) (Figure 2b). In addition, all doses of systemic tapentadol inhibited pre‐conditioned mechanically evoked neuronal firing but this effect did not reach significance (two‐way ANOVA: *p* > 0.05 for all doses and forces tested). This indicates that when the endogenous inhibitory system serving DNIC is functional, as is the case in sham controls, increased synaptic levels of noradrenaline and MOR agonism have a limited effect on the level of inhibition induced by DNIC.

### 3.3 The actions of pregabalin in MIA animals

In MIA animals, pregabalin significantly inhibited pre‐conditioned mechanically evoked neuronal responses for all mechanical stimulations tested (two‐way ANOVA; 8 g: *p* < 0.05, 26 g: *p* < 0.01, 60 g: *p* < 0.001, *n* = 6) (Figure 3a; See Supporting Information Table S2). As pregabalin is preferentially effective in states of central neuronal dysregulation, pregabalins ability to significantly reduce mechanically evoked neuronal firing may be indicative of central sensitization or a neuropathic component, which are sufficient conditions for pregabalin to become efficacious. However, pregabalin proved ineffective at restoring DNIC in MIA animals as there was no reduction in mechanically evoked neuronal firing with a noxious conditioning ear pinch, indicating pregablin cannot restore descending inhibitory tone (Figure 3a).

In sham controls, pregabalin did not inhibit pre‐conditioned mechanically evoked neuronal firing or affect the level of neuronal inhibition induced by DNIC (two‐way ANOVA: *p* > 0.05 for all forces tested, *n* = 6) (Figure 3b). The magnitude of neuronal inhibition was 48%, 38% and 34% before drug application, and 38%, 37% and 33% following systemic pregabalin for 8, 26 and 60 g stimulations, respectively. As the degree of neuronal inhibition induced by DNIC was comparable before and after pregabalin administration, it indicates that pregabalin has a limited effect on the endogenous inhibitory system in sham controls.

The α2δ1 subunit of VGCCs is upregulated and contributes to spinal hyperexcitability in states of neuropathy; therefore, the mRNA expression of the α2δ1 subunit was investigated in ipsilateral lumbar DRGs and dorsal horn following MIA injection (Li et al., 2006). Interestingly, there was no increase in the mRNA expression of the α2δ1 subunit in either the lumbar DRGs or dorsal horn in MIA animals compared to sham controls (Independent samples *t* test; *p* < 0.05, MIA *n* = 4, Sham *n* = 4). This indicates that the efficacy of pregabalin in MIA animals is not due to an upregulated expression of the α2δ1 subunit and subsequent increase in trafficking of VGCCs to the plasma membrane (Bauer et al., 2009).

### 3.4 A combination therapy of tapentadol and pregabalin in MIA animals

Tapentadol dose‐dependently inhibited pre‐conditioned mechanically evoked neuronal responses and restored neuronal inhibition induced by DNIC in MIA animals; however, tapentadol was only effective at reducing pre‐conditioned neuronal responses for noxious 26 and 60 g forces. Tapentadols lack of efficacy to inhibit pre‐conditioned neuronal responses to innocuous stimulations may indicate that tapentadol would be ineffective at relieving allodynia. Meanwhile, pregabalin proved effective at inhibiting pre‐conditioned mechanically evoked neuronal responses to all stimulations tested in MIA animals but did not restore neuronal inhibition induced by DNIC. Therefore, a combination therapy of tapentadol and pregabalin was assessed in MIA animals to investigate if this may be an effective approach for inhibiting pre‐conditioned neuronal responses to both innocuous and noxious stimulations, whilst also restoring descending inhibitory tone.

The combined tapentadol and pregabalin treatment revealed DNIC as a significant reduction in neuronal firing was induced with a noxious conditioning ear pinch in response to 26 and 60 g at both dose combinations (two‐way ANOVA; 1 and 10 mg/kg: 8 g: *p* > 0.05, 26 g: *p* < 0.05, 60 g: *p* < 0.001, 2 and 5 mg/kg: 8 g: *p* > 0.05, 26 g: *p* < 0.01, 60 g: *p* < 0.001, *n* = 6 for both doses) (Figure 4a). Both dose combinations also significantly inhibited pre‐conditioned neuronal responses for all mechanical stimulations tested (two‐way ANOVA: 1 and 10 mg/kg: 8 g: *p* < 0.01, 26 g: *p* < 0.05, 60 g: *p* < 0.001, 2 and 5 mg/kg: 8 g: *p* < 0.05, 26 g: *p* < 0.001, 60 g: *p* < 0.001, *n* = 6; See Supporting Information Table S3). The magnitude of neuronal inhibition with a conditioning noxious ear pinch was 41%, 34% and 26% following 1 mg/kg tapentadol and 10 mg/kg pregabalin, and 17%, 28% and 20% following 2 mg/kg tapentadol and 5 mg/kg pregabalin, which is similar to the magnitude of inhibition observed in sham controls.

In sham controls, both dose combinations had a limited effect on the degree of neuronal inhibition induced by DNIC (Figure 4b). Interestingly, the combined systemic dose 1 mg/kg tapentadol and 10 mg/kg pregabalin did not significantly inhibit pre‐conditioned mechanically evoked neuronal firing for any mechanical stimulations tested (two‐way ANOVA: *p* < 0.05 for all weight, *n* = 6). This may indicate that without sufficient conditions for pregabalin to be effective, the low dose of tapentadol alone is not enough to inhibit pre‐conditioned neuronal firing. However, the combined 2 mg/kg tapentadol and 5 mg/kg pregabalin dose does significantly inhibit the pre‐conditioned neuronal firing for the 60 g mechanical stimulation (two‐way ANOVA: 8 g and 26 g: *p* > 0.05, 60 g: *p* < 0.001, *n* = 5). This indicates that although pregabalin is not effective in sham controls, the higher dose of tapentadol is sufficient to inhibit neuronal firing to the most noxious mechanical stimulations.

## 4 DISCUSSION

This study agrees with pre‐existing evidence that using in vivo electrophysiological techniques allows for consistent and reliable measurement of DNIC responses and offers the opportunity to assess the functionality of descending controls in animal models of chronic pain (Bannister et al., 2015; Le Bars et al., 1979). It is important animal studies are translated to the clinic to increase our understanding of mechanisms involved in pain pathways and thus recognize potential therapeutic agents. This study provides a good opportunity for forward translation as measuring CPM responses in the clinic can also provide information on a patients endogenous inhibitory system (Edwards, Ness, Weigent, & Fillingim, 2003). Indeed, patients with a less efficient CPM before surgery were more susceptible to developing post‐operative chronic pain following surgery, indicating that assessing CPM responses can provide crucial information on a patient's physiology (Yarnitsky et al., 2008). We have demonstrated that DNIC are absent in MIA animals, and similarly a study found patients with severe knee OA pain had significantly less DNIC than healthy controls, indicating relevance of our study to the clinic (Arendt‐Nielsen et al., 2010).

All doses of tapentadol restored neuronal inhibition induced by a noxious conditioning ear pinch to similar levels observed in sham controls. However, tapentadol had a limited effect on the magnitude of neuronal inhibition induced by DNIC in sham controls, indicating that when the descending controls are functional, increasing the synaptic availability of noradrenaline and MOR agonsim have limited further effects on neuronal inhibition. In addition, tapentadol dose‐dependently inhibited pre‐conditioned mechanically evoked neuronal firing in MIA animals although this was only significant for the most noxious stimulation of 60 g indicating tapentadol may be less effective at relieving allodynia.

The MOR and NRI contributions to tapentadols mechanism of action at the level of the spinal cord have been assessed in a spinal nerve ligation (SNL) model of neuropathy (Bee, Bannister, Rahman, & Dickenson, 2011; Schroder, Vry, Tzschentke, Jahnel, & Christoph, 2010; Tzschentke et al., 2007). All studies found the inhibitory actions of tapentadol were most effectively blocked by the MOR antagonist naloxone in sham controls. However, in SNL animals the analgesic efficacy of tapentadol was strongly reduced by the α2‐adrenoceptor antagonists yohimbine or atipamezole, but only moderately reduced by naloxone (Bee et al., 2011; Schroder et al., 2010; Tzschentke et al., 2007). The authors proposed that tapentadol utilizes a predominant opioid mechanism to mediate inhibition in control animals, which shifts to a predominant noradrenergic inhibitory mechanism following central hyperexcitability or dysregulation in descending controls. Therefore, the efficacy of tapentadol at inhibiting pre‐conditioned mechanically evoked neuronal firing in sham animals is likely due to a predominant opioid mechanism. Meanwhile, the efficacy of tapentadol at restoring neuronal inhibition induced by DNIC in MIA animals, even at low doses, is likely due to its ability to increase the synaptic content of noradrenaline, which can subsequently mediate neuronal inhibition through activating α2‐adrenoceptors.

Tapentadol provided significant pain relief and activated abolished CPM responses in patients with diabetic polyneuropathy, indicating that tapentadols analgesic efficacy was dependent on its ability to activate descending inhibitory pathways (Niesters et al., 2014). Similarly, in a cohort of patients with painful diabetic neuropathy, baseline CPM was correlated with the efficacy of the serotonin–noradrenaline reuptake inhibitor duloxetine, such that duloxetine was most effective in patients with a less efficient CPM (Yarnitsky, Granot, Nahman‐Averbuch, Khamaisi, & Granovsky, 2012). Taken together, this indicates that testing CPM responses has great potential for providing valuable insights into how likely a patient is to respond to an analgesic that restores descending inhibitory tone. The common pharmacological mechanism shared by tapentadol and duloxetine is their function as NRIs. Therefore, our findings coupled with these human studies indicate that tapentadol will prove most effective in cases where the endogenous inhibitory system is dysfunctional and confirm pre‐existing evidence that DNIC can be activated through restoring descending noradrenergic inhibitory pathways, due to the subsequent actions of noradrenaline at α2‐adrenoceptors in the spinal cord (Bannister et al., 2015, 2017).

Pregabalin proved effective significantly inhibiting all pre‐conditioned mechanically evoked neuronal responses in MIA animals, yet was ineffective in shams, which agrees with previous studies that gabapentinoid drugs are more effective at modulating spinal nociceptive transmission in animal models with central neuronal hyperexcitability (Bee & Dickenson, 2008; Stahl et al., 2013). Gabapentinoid drugs bind with high affinity to the α2δ1 subunit of VGCCS, and several studies indicate that α2δ1 subunit expression levels are increased in both ipsilateral DRGs and the dorsal horn following neuropathy, supporting the concept that the α2δ1 subunit is the molecular target for mediating an analgesic effect (Bauer et al., 2009; Luo et al., 2001; Newton, Bingham, Case, Sanger, & Lawson, 2001). The upregulated α2δ1 subunit increases trafficking of VGCCs to neuronal plasma membranes, which subsequently increases calcium influx and neurotransmitter release, therefore a proposed mechanism for the antinociceptive function of pregabalin is that through binding to the α2δ1 subunit it prevents trafficking of VGCCs and inhibits the enhanced release of neurotransmitters (Bauer et al., 2009; Hendrich et al., 2008). However, given the time required for axonal trafficking events, it is unlikely this proposed mechanism is responsible for pregabalins acute effects as observed in this study (Patel & Dickenson, 2016). Furthermore, as we found no increase in the mRNA expression of the α2δ1 subunit, it may suggest there is no neuropathic component to our MIA model and that pregabalin is functioning through an alternate mechanism.

Despite the α2δ1 subunit being the target of the analgesic actions of gabapentinoids, its upregulation is not always crucial for gabapentinoid drugs to have effective analgesic actions (Suzuki et al., 2005). The development of central sensitization and the subsequent enhanced activity of descending brainstem facilitations acting on spinal 5‐HT_3_ receptors has been demonstrated to create the necessary conditions for gabapentinoid drugs to mediate analgesia (Suzuki et al., 2005). Interestingly, when the spino‐bulbo‐spinal loop was disrupted in SNL rats, through ablation of spinal NK1 projection neurons or μ‐opioid receptor‐expressing neurons in the RVM with the neurotoxin saporin, gabapentinoids could no longer inhibit the excitability of WDR neurons in the dorsal horn (Bee & Dickenson, 2008; Suzuki et al., 2005). Remarkably, when a 5‐HT_3_ receptor agonist was applied spinally in naive rats, pregablin could inhibit spinal neurons even in the absence of injury (Suzuki et al., 2005). Furthermore, the intrathecal administration of the 5‐HT_3_ receptor antagonist ondansetron attenuated tactile allodynia and thermal hyperalgesia in SNL mice and transgenic mice overexpressing the α2δ1 subunit (Chang, Chen, Sandhu, Li, & Luo, 2013). The lack of DNIC in MIA animals strongly suggests enhanced descending facilitatory serotonergic controls are acting at 5‐HT_3_ receptors in the spinal cord (Bannister et al., 2015). Together, this suggests that despite no upregulation of the α2δ1 subunit, the activation of pre‐synaptic 5‐HT_3_ receptors in the dorsal horn, due to enhanced descending facilitations, creates adequate conditions for pregabalin to inhibit calcium currents and subsequently prevent neurotransmitter release in MIA animals. Despite pregabalin proving effective at inhibiting pre‐conditioned mechanically evoked neuronal responses, it did not restore the DNIC system in MIA animals. There is no evidence to indicate that pregabalin restores the balance in facilitatory and inhibitory descending controls, and therefore it cannot reinstate the DNIC system in MIA animals. Furthermore, this finding is in agreement with a human study, where pregabalin proved an effective analgesic in patients with chronic pancreatitis, yet did not restore CPM responses (Bouwense et al., 2012).

During the development of OA, multiple concurrent mechanisms alter nociceptive transmission and result in hypersensitivity (Schaible & Grubb, 1993). Therefore, tackling multiple mechanisms with combination therapy may not only provide superior analgesia but agents may also be used at lower doses such that the adverse side effects associated with each drug are minimized (Gilron, Jensen, & Dickenson, 2013). Indeed, although tapentadol has a much lower affinity for the MOR compared to traditional opioids, it would still be preferable to use lower doses in patients to avoid the adverse side effects associated with opioid analgesia (Tzschentke et al., 2007, 2012). In this instance, we found that a combination therapy of pregabalin and tapentadol provided effective inhibition of pre‐conditioned neuronal responses whilst also restoring neuronal inhibition induced by DNIC. Therefore, through their respective mechanisms, this combination used at low doses may be a feasible approach for both restoring descending inhibitory tone but also for tackling central sensitization and hypersensitivity at the level of the spinal cord.

The advancement in the understanding of peripheral and central molecular mechanisms behind OA pain, coupled with the shift towards personalized medicine in the clinic, means patients should be more thoroughly diagnosed to understand their individual pain phenotype in order to prescribe the most effective analgesic. This study makes a strong case for investigating patients CPM responses, to identify if analgesic agents that restore descending inhibitory tone would be beneficial, while patients with central neuronal hyperexcitability may benefit from gabapentinoids, to tackle their OA pain. Furthermore, this study makes a case for combination therapies when patients report with multiple symptoms, which may tackle the issues of limited efficacy or dose‐limiting side effects associated with monotherapy.

## CONFLICT OF INTEREST

The authors have no conflict of interest to declare.

## AUTHOR CONTRIBUTIONS

Stevie Margaret Lockwood: concept and design, analysis and drafting of the article; Anthony H. Dickenson: concept and design, final approval of the article.
