# Supplementary material for: A combination pharmacotherapy of tapentadol and pregabalin to tackle centrally driven osteoarthritis pain
Source: Eur J Pain. 2019 Mar 22;23(6):1185–95. doi: 10.1002/ejp.1386 (PMC6618140; doi:10.1002/ejp.1386)
Supplement: Supplementary file 1 [file EJP-23-1185-s001.docx]

Table S1. Cohen’s D value for Tapentadol pre-conditioned pairwise comparisons

| Comparison | Cohen’s D |
| --- | --- |
| **MIA animals: Tapentadol effect on pre-conditioned responses** | |
| 8g: 1mg Tapentadol | 0.50 |
| 26g: 1mg Tapentadol | 0.32 |
| 60g: 1mg Tapentadol | 0.68 |
| 8g: 2mg Tapentadol | 0.05 |
| 26g: 2mg Tapentadol | 0.85 |
| 60g: 2mg Tapentadol | 1.74 |
| 8g: 5mg Tapentadol | 0.48 |
| 26g: 5mg Tapentadol | 1.82 |
| 60g: 5mg Tapentadol | 2.43 |
| **Sham animals: Tapentadol effect on pre-conditioned responses** | |
| 8g: 1mg Tapentadol | 0.40 |
| 26g: 1mg Tapentadol | 0.20 |
| 60g: 1mg Tapentadol | 0.52 |
| 8g: 2mg Tapentadol | 0.94 |
| 26g: 2mg Tapentadol | 1.45 |
| 60g: 2mg Tapentadol | 1.70 |
| 8g: 5mg Tapentadol | 0.03 |
| 26g: 5mg Tapentadol | 0.45 |
| 60g: 5mg Tapentadol | 0.87 |

**Table S2.** Cohen’s D value for Pregabalin pre-conditioned pairwise comparisons

| Comparison | Cohen’s D |
| --- | --- |
| **MIA animals: Pregabalin effect on pre-conditioned responses** | |
| 8g: 10mg Pregabalin | 1.21 |
| 26g: 10mg Pregabalin | 1.80 |
| 60g: 10mg Pregabalin | 2.39 |
| **Sham animals: Pregabalin effect on pre-conditioned responses** | |
| 8g: 10mg Pregabalin | 0.27 |
| 26g: 10mg Pregabalin | 0.27 |
| 60g: 10mg Pregabalin | 0.18 |

**Table S3.** Cohen’s D value for Tapentadol and Pregabalin pre-conditioned pairwaise comparisons

| Comparison | Cohen’s D |
| --- | --- |
| **MIA animals: Tapentadol and Pregabalin effect on pre-conditioned responses** | |
| 8g: 1mg Tapentadol + 10mg Pregabalin | 0.83 |
| 26g: 1mg Tapentadol +10mg Pregabalin | 0.64 |
| 60g: 1mg Tapentadol + 10mg Pregabalin | 0.64 |
| 8g: 2mg Tapentadol + 5mg Pregabalin | 0.67 |
| 26g: 2mg Tapentadol + 5mg Pregabalin | 1.75 |
| 60g: 2mg Tapentadol + 5mg Pregabalin | 2.95 |
| **Sham animals: Tapentadol and Pregabalin effect on pre-conditioned responses** | |
| 8g: 1mg Tapentadol + 10mg Pregabalin | 0.12 |
| 26g: 1mg Tapentadol + 10mg Pregabalin | 0.04 |
| 60g: 1mg Tapentadol + 10mg Pregabalin | 0.57 |
| 8g: 2mg Tapentadol + 5mg Pregabalin | 0.65 |
| 26g: 2mg Tapentadol + 5mg Pregabalin | 0.35 |
| 60g: 2mg Tapentadol + 5mg Pregabalin | 1.95 |
